# Editorial Special Issue: Neuronus

**DOI:** 10.5709/acp-0194-4

**Published:** 2016-12-31

**Authors:** Rob H. J. Van der Lubbe, Michał Kuniecki

**Affiliations:** 1 Cognitive Psychology and Ergonomics, University of Twente,The Netherlands; 2 Department of Cognitive Psychology, University of Finance and Management in Warsaw, Poland.; 3 Psychophysiological Laboratory of the Jagiellonian University, Kraków, Poland

**Keywords:** working memory, brain oscillations, wavelet analyses, ERPs, motor imagery, motor preparation, SNARC effect, physical pain, emotional pain, EEG, ERN, Pe, SSVEP, gestures, memory retrieval, P3b, S-R link hypothesis, oddball task, complete locked-in patients, brain plasticity

## Abstract

This special issue of the 12th volume of Advances in Cognitive Psychology is devoted to the Neuronus conference that took place in Kraków in 2015. In this editorial letter, we will focus on a selection of the materials and some follow-up research that was presented during this conference. We will also briefly introduce the conference contributions that successfully passed an external reviewing process.

## Introduction

The Neuronus conference in 2015 took place from the 17th until the 19th of April and was hosted by the Jagiellonian University in Kraków. Neuronus has been supported by both the International Brain Research Organization (IBRO) as well as the International Research Universities Network (IRUN), a consortium of European universities founded by the Radboud University from Nijmegen in the Netherlands.

Two-hundred and sixty-one authors were registered. There were seven plenary lectures, and 16 parallel sessions that consisted of 68 talks in total. Moreover, in three different poster sessions, 236 posters were presented. It is of course impossible to get a full impression of all that was presented and discussed during the conference and most of the very interesting topics are not covered in our special issue. Nevertheless, we hope that the current selection at least reflects the inspiring vibe of the conference.

 On Friday, April 17, the session “Advanced EEG Signal Analyses” took place. Two presentations of this session resulted in two of the articles that are published in this special issue. The first talk by Boris Gutkin considered the role of various types of oscillations of electrophysiological brain responses underlying working memory, which culminated in the article by Dipoppa, Szwed, and Gutkin ( 2016). In this broad review paper, a major update of current knowledge on working memory is presented, in which oscillations of electrophysiological brain activity are interpreted in terms of the specific operations that are needed for a successful functioning of working memory. One of the reviewers of this paper (Munk, 2016), was invited to write a commentary letter. In this letter, he emphasizes that the novelty of the article of Dipoppa et al. lies in its grounding of the various results in a systems-neuroscience approach. The other talk in this session by the first author of this Introduction focused on the extra information that can be extracted from the electroencephalogram (EEG) when performing wavelet analyses on the raw EEG and of event-related potentials (ERPs). This resulted in the article by Van der Lubbe, Szumska, and Fajkowska (2016). In this article, it was examined whether the P1 ERP component can be understood as phase-locked alpha activity. Additionally, it was explored whether the majority of EEG activity after a visual stimulus is evoked or induced, or simply a continuation of prestimulus activity. Finally, the authors tested whether early ERP components are due to a phase reset of ongoing oscillations. Results revealed that the P1 is related to alpha activity, the majority of EEG activity appears not to be evoked, and it seems unlikely that early ERP components are due to a phase reset. 

 The poster session on Friday included 77 posters. One poster presented by Jagna Sobierajewicz resulted in an article that is included in the special issue, too ( Sobierajewicz, Szarkiewicz, Przekoracka-Krawczyk, Jaśkowski, & Van der Lubbe, 2016). Sobierajewicz et al. (2016) examined to what extent learning of a sequence of finger movements by motor imagery can replace learning by motor execution. The data revealed that a substantial amount of physical practice next to practice by motor imagery was required to obtain a comparable speed as after full physical practice. Interestingly, the data additionally suggested that motor preparation and motor imagery induced comparable learning effects. Another article that was related to an earlier Neuronus meeting is the article by Gut and Staniszewski (2016). This article is the outcome of a project that was developed together with Piotr Jaśkowski, the founding editor of Advances in Cognitive Psychology (ACP), who passed away in 2011. This study shows that the spatial numerical association of response codes (SNARC) effect also affects the memorization and retrieval of numbers. 

 On Saturday, one morning session was devoted to “Pain and the Brain”. This session started with a talk by André Mouraux, who published an impressive amount of articles in this field of research. A recent article co-authored by him that relates to the topic that he presented is by Iannetti, Salomons, Moayedi, Mouraux, and Davis (2013). In this article, the authors focused on the question whether the notion that “physical pain and social distress share a common neurobiological substrate”(Eisenberger, Lieberman, & Williams, 2003) can be maintained after a critical evaluation. Shortly after the article by Eisenberger et al. (2003), it was also proposed that “social exclusion is experienced as painful because reactions to rejection are mediated by aspects of the physical pain system” (MacDonald & Leary, 2005). Thus, the question is whether shared activation for different types of stimuli (nociceptive, i.e., involving activation of the pain pathways, or emotional) can really be interpreted as a common experience. Iannetti et al. first pointed out that there is a logical problem with this claim, as the authors use reversed inferencing. Secondly, the activation of brain areas like the anterior cingulate cortex and the insula has also been observed in the case of nonnociceptive stimuli like auditory stimuli, and these activations are not accompanied by a pain experience. Finally, recent, more detailed approaches using multivariate pattern analysis (MVPA) of functional magnetic resonance imaging (fMRI) data actually pointed to spatially distinct patterns of brain activity in the case of social and physical pain. Thus, emotional and physical pain seem to involve different mechanisms, and the idea that social distress is quite comparable to physical distress can no longer be maintained. 

 During the poster session on Saturday, Katharina Paul and colleagues presented results of their EEG experiment, entitled “Happy and blind to response errors? New insights from error-related event-related-brain potentials.” Their data were later published by Paul, Walentowska, Bakic, Dondaine, and Pourtois (2016). In this paper, they revealed that unlike negative mood, positive mood does not influence the early automatic EEG component reflecting error detection (ERN), but that it influences the later awareness-related positive going component, dubbed *Pe*. The authors concluded that positive mood does not interfere with cognitive control, such as error monitoring, but instead triggers adaptive changes that cause the organism to transiently downplay the impact of negative events. 

 A later session that day focused on affective neuroscience. In this session, Matthias Wieser examined the processing of threat-related social cues by using steady-state visually evoked potentials (SSVEPs). This presentation seems related to an article that was published in 2014 (Wieser, Flaisch, & Pauli, 2014). In this paper, the authors focused on the conditioning of neutral faces by pairing them with affective nonverbal gestures (a negative raised middle finger, a positive thumbs up, or a neutral pointing gesture) in an acquisition phase. The gestures were presented directly after presenting the facial stimuli. Each face was presented within a flickering background at a frequency of 12 Hz. This enables to determine face-specific processing in the EEG by computing the SSVEPs. Interestingly, in the acquisition phase, the SSVEP amplitude was larger for the faces that were paired with the negative gesture than for the faces related with the neutral gesture, while no clear differences were observed in comparison with the positive gesture. These findings were also reflected in subjective valence ratings after the acquisition phase, as faces paired with negative gestures were rated as more unpleasant than faces related with neutral and positive gestures. These findings suggest that social conditioning by gestures alters processing in the visual cortex, which may be due to modulations from subcortical areas like the amygdala. Thus, ecologically valid social cues like a raised middle finger have a direct effect on the processing of associated facial stimuli in visual cortex. 

 Another session on Saturday was entitled “Aged 50 and still to be explored yet: The P3 component of event-related potentials.” In this session, one presentation held by Siri-Maria Kamp resulted in the article by Kamp, Bader, and Mecklinger (2016) that is included in the present special issue, too. In this article, the authors focused on the contributions of familiarity and recollection to associative retrieval of word pairs depending on task instructions during the encoding phase. They examined relatively early frontal and later parietal modulations that may actually be related to different subcomponents of the P3, although this was not explicitly stated in their manuscript. They argued that their results accord with prior studies in which it was shown that successful retrieval may occur without hippocampal involvement (for more details, see Kamp et al., 2016). Rolf Verleger chaired this session and gave a talk with the title “Bridging events and action: P3b reflects activation of stimulus-response links.” In 2016, three articles were published that are all closely related to this talk (Verleger, Grauhan, & Śmigasiewicz, 2016a , 2016 b; Verleger & Śmigasiewicz, 2016). The study by Verleger et al. ( 2016a) revealed that oddball effects on the P3b component, the enhanced P3 component for rare as compared to frequent stimuli, vary depending on the extent by which stimuli can be linked to a specific response. Rare stimuli evoked the largest P3b component when the response to this stimulus was specified beforehand by a preceding stimulus-response (S-R) mapping cue. In contrast, the P3b for rare stimuli was much smaller (although not absent) when the S-R mapping at the time of stimulus presentation was unknown, as the cue followed the rare stimuli. These findings can by and large be interpreted along the lines of the *S-R link* hypothesis, according to which the P3b component reflects the reactivation of an S-R link. Interestingly, in the second article (Verleger et al., 2016b), the focus was on whether the P3 is a reflection of a tactical (directly relevant) or a more strategic (relevant for the long term) process. Oddball tasks were examined with rare/frequent Go or NoGo trials and *Choice Response* trials. The P3 components observed in these tasks were decomposed, by the residue-iteration decomposition (RIDE) technique, into a stimulus-related (S-P3), a central-processing related (C-P3), and a response-related (R-P3) part. The R-P3 occurred together with fast responses to frequent stimuli, while the C-P3 coincided with the responses to rare stimuli. Thus, the C-P3 does not seem relevant for frequent responses, which might mean that this component reflects the reactivation of an S-R link. It was concluded that these results fit with a tactical view of the P3, as responses to frequent stimuli rely on a fast or even an ultra-fast path that does not require a thorough stimulus analysis. In the third article (Verleger & Śmigasiewicz, 2016), which was published earlier this year in our journal, the question was addressed whether the oddball P3 reflects unexpectedness of rare stimuli, which is often taken for granted. A task was developed in which participants had to predict what stimulus (frequent/rare) they expected to occur on a specific trial. Importantly, in contrast with the aforementioned idea, the increased P3 for rare stimuli was completely unaffected by expectancies. In combination with several other aspects of their results, the authors concluded that these findings seem mostly in line with the idea that the P3 reflects subjective relevance. Nevertheless, there also seems to be some room to fit these results with the S-R link hypothesis. For example, there might be some rigidity in implementing the new S-R link, as is demonstrated by task-switching experiments. This aspect might explain why the P3 for predicted rare stimuli remains large. 

 On Sunday morning, Niels Birbaumer gave a highly interesting plenary lecture. An article that seems related to this lecture appeared in *Brain Topography* (Birbaumer, Gallegos-Ayala, Wildgruber, Silvoni, & Soekadar, 2014). In this article, it is concluded that until now, little progress has been made in improving communication with patients in a complete locked-in state (CLIS). Earlier attempts to communicate were largely based on imagery and operant conditioning procedures. It was argued that the failure to communicate may be related to the *extinction-of-thought* hypothesis. Studies demonstrated that if a behavioral response is emitted independent from an intention, then the awareness of a relation between a thought and a reward may vanish. An alternative approach was proposed that is based on classical semantic conditioning of autonomic or brain responses, as this method also seemed to work in the completely paralyzed rat and it does require only minimal attentional resources and effort. Procedures were developed to classify EEG oscillations, ERPs, and near-infrared spectroscopy (NIRS) signals associated with “yes” or “no” responses. A study was reported in which communication on the basis of NIRS resulted in 72% up to 100% correct classifications, which indeed suggests that semantic classical conditioning may result in stable communication with CLIS patients. 

 In the afternoon, a session focused on language and semantic processing took place. The talk of Marcin Szwed relates to the paper of Siuda-Krzywicka et al. (2016) that appeared in *eLIFE*. In this article, the authors thoroughly explored the possibility whether the visual cortex of the normal adult brain may also be recruited when learning Braille reading, comparable to the reorganized visual cortex of the blind for the processing of tactile and auditory stimuli. Participants took part in a very intensive nine-month Braille-reading course. After the course, fMRI results revealed that tactile reading increased activity in the visual cortex, including the visual word form area. Importantly, disruption of activity in this area by transcranial magnetic stimulation (TMS) interfered with participants’ tactile reading accuracy. These results indicate that a major reorganization of the cortex can occur after learning a complex skill and that our visual cortex can be recruited for nonvisual purposes. These findings further question the idea that the human brain is divided in completely separate areas devoted for vision, tactile perception, and so forth, as intensive training may modify the sensory-division of labor in human brains. 

In conclusion, the Neuronus meeting in Kraków in 2015 showed highly interesting talks on a variety of topics. We want to thank all the authors, reviewers, and involved editors for their help, which led to a very interesting special issue with high-quality papers. This year, the decision was made by the organizers that Neuronus will become a biannual meeting, so the next meeting will probably take place at the end of April 2018 in Kraków. An additional special issue related to the Neuronus conference from this year may come out next year. For more details and updates on Neuronus conferences see http://neuronusforum.pl
